# Editorial: Negotiating borders: body politics and artivism in migration

**DOI:** 10.3389/fsoc.2026.1984093

**Published:** 2026-10-15

**Authors:** Paula Guerra, Júlia Almeida de Mello, Sofia Sousa

**Affiliations:** 1 University of Porto, Porto, Portugal; 2 Federal University of Espírito Santo, Vitoria, Brazil

**Keywords:** artivism, bodies, gender, Global South, migration

Over recent decades, the body has increasingly emerged as a privileged site of political negotiation, particularly where it intersects with gender, migration and artistic creation. This Research Topic, published in the Migration and Society section of Frontiers in Sociology, is situated precisely at this intersection of migration, corporeality and artivism.

This reading is grounded in a broader analytical and epistemological program. Following decolonial and intersectional feminist epistemologies (Quijano, 2007; Maldonado-Torres, 2007; Crenshaw, 1991; Lugones, 2010), the body is here understood as a site where race, gender, class and territory are co-constituted, and where the afterlives of coloniality continue to determine who is granted mobility and who remains confined within physical, symbolic or institutional borders. Diaspora and displacement are accordingly never merely descriptive categories: they name an unfinished process of attachment and re-attachment (Brah, 2005; Clifford, 1994) through which bodies inscribe, resist and reconfigure the traces of dispossession, converting vulnerability into a resource for creation, memory and voice. This reading also finds support beyond migration strictly defined. Elsewhere, in examining the trajectory of the band Fulô do Sertão and the emergence of Feminejo in Northeast Brazil, we have shown how the rural female body becomes a vehicle of contestation against patriarchal traditionalism, reconfiguring gender, territory and voice through a do-it-yourself ethos of artivism (Guerra, 2024). Situated in the Global South, and largely absent from Anglo-American scholarship on popular music and the arts, such cases corroborate the wider argument developed across the present Research Topic: that artistic practice—whether performative, literary, musical or visual—offers migrant, racialised, gendered and dissident bodies a means of claiming voice precisely where formal political channels remain closed to them. It is against this backdrop that the five contributions gathered here should be read.

Studies such as Patteri (2024) demonstrate that art and its corporeal manifestations, in relation to migrant women, constitute spaces of subversion and resistance against symbolic borders, but also against physical borders that are increasingly closed and deterministic. However, the coordinators of this Research Topic contend that research remains scarce on the connections between dynamics of social (dis)order and the everyday practices of art and life at the threshold of these borders. The five articles gathered here respond to this gap from distinct disciplinary perspectives, drawing variously on postcolonial literary studies, visual sociology and art history. Ultimately, they converge on a single question: how the body, placed in plural situations of displacement, gender or dissidence, becomes a political instrument for claiming voice and for individual and collective self-assertion in the public sphere.

The first article, by Nila and Mukherjee, entitled “*From Zenana to courtroom: the politics of genre and gender in Sujata Massey's historical crime fiction*,” analyses the novel *A Murder on Malabar Hill* (2018) considering the concept of hybridity and the third space. Through the protagonist Perveen Mistry, a Parsi lawyer, woman and figure embedded in a colonial context, the authors show how postcolonial detective fiction shifts the rational European detective toward a figure of cultural mediation, situated between British law and indigenous legal systems. Also mobilizing classical authors of urban sociology such as Lefebvre (1991) and Soja (1996), as well as Haraway's (1988) notion of situated knowledges, the article interprets the city of Bombay as a third space in which justice comes to be a kind of situated negotiation.

The second article, by Guerra “*Migrations, arts, and bodies: the* Silhouette in *multiple shadows of Rubiane Maia*,” focuses on Rubiane Maia, a Brazilian artist based in the United Kingdom. Combining a biographical approach with categorical and content analysis of a semi-structured interview and of two performances—Book-Performance and Speirein—the author shows how Maia transforms the biographical rupture brought about by migration and motherhood into a corporeal archive of memory. The article brings together theoretical frameworks on coloniality (Quijano, 2007; Maldonado-Torres, 2007), decolonial intersectionality (Crenshaw, 1991; Lugones, 2010) and diaspora studies (Brah, 2005; Clifford, 1994), demonstrating how the vulnerability of the Black migrant woman's body is converted into a creative force that enters into a wider critical dialogue with collective memory.

In turn, Mello, in “*Freak to queer bodies: affirming the grotesque in contemporary art*,” traces a genealogy between the bodies exhibited in nineteenth-century freak shows and the queer bodies in the artistic productions of Fernanda Magalhães and Laura Aguilar. Drawing on the Bakhtinian grotesque (Bakhtin, 1984), Goffman's (1963) notion of stigma and Foucauldian biopolitics (Foucault, 1980), the author argues that bodily difference, more specifically fatness, continues to function as a border that defines normality, and that the artistic appropriation of the grotesque constitutes a political strategy of resistance and visibility.

The fourth article, by Sousa, “*New forms of artivism in relation to contemporary societies: Tuty Moreno Campos's soft artivism*,” proposes the concept of soft artivism, developed from the work of the Argentine artist Tuty Moreno Campos, currently resident in Mexico. Drawing on Warner (2021) and on Bovenschen's (1977) tradition of feminist aesthetics, the author argues that Campos's artivism—in the performances *Vaciarce* (2019) and Á*rido* (2020)—in relation to the legacy of feminist movements, shifts the focus away from the public confrontation typically associated with traditional activist practice, toward a field of intimate and sensitive transformation, in a register of informality, experimentation and accessibility that engages in dialogue with Butler's (2013) performativity.

Finally, the article by Alwulaii, “*Body, gender, race and fatness in social studies: gap and future*,” offers a critical framework that runs transversally across the four preceding investigations. Drawing on Bourdieu (1984), Foucault (1980), and Shilling (2012), four structural gaps in the sociology of the body are identified, namely the over-representation of the white female body, the subalternisation of other races and ethnicities, the geographical concentration on Western societies, and the reduction of fatness to a medical problem to the detriment of its reading as a social phenomenon. The article thereby reinforces the relevance of investigations such as those brought together in this Research Topic, insofar as they shift the gaze toward the Global South and toward bodies that have been historically marginalized by dominant academic and scientific literature.

Read together, these five contributions substantively confirm that the migrant, female, racialized, queer, artist body constitutes a terrain of symbolic and political contestation. From colonial detective fiction to contemporary performance, from grotesque imagery to soft artivism, the articles brought together here demonstrate that art effectively functions as a device for translation between competing systems of knowledge, and that artivism—as practice, way of life and mode of assertion—remains a fundamental instrument for the negotiation of borders, identities and social inequalities. A challenge nonetheless remains for future research: deepening intercultural and transnational comparisons that would allow us to understand how these dynamics of body, gender. and migration are articulated beyond the contexts studied here—an invitation that this Research Topic leaves open to the scientific community.
